# Natural Killer (NK) Cell Education Differentially Influences HIV Antibody-Dependent NK Cell Activation and Antibody-Dependent Cellular Cytotoxicity

**DOI:** 10.3389/fimmu.2017.01033

**Published:** 2017-08-24

**Authors:** Nicole F. Bernard, Zahra Kiani, Alexandra Tremblay-McLean, Sanket A. Kant, Christopher E. Leeks, Franck P. Dupuy

**Affiliations:** ^1^Research Institute of the McGill University Health Centre, Montreal, QC, Canada; ^2^Division of Experimental Medicine, McGill University, Montreal, QC, Canada; ^3^Chronic Viral Illness Service, McGill University Health Centre, Montreal, QC, Canada; ^4^Division of Clinical Immunology, McGill University Health Centre, Montreal, QC, Canada

**Keywords:** natural killer cells, antibody-dependent cellular cytotoxicity, antibody-dependent natural killer cell activation, natural killer cell education, CD16, inhibitory natural killer cell receptors, non-neutralizing antibodies, broadly neutralizing antibodies

## Abstract

Immunotherapy using broadly neutralizing antibodies (bNAbs) endowed with Fc-mediated effector functions has been shown to be critical for protecting or controlling viral replication in animal models. In human, the RV144 Thai trial was the first trial to demonstrate a significant protection against HIV infection following vaccination. Analysis of the correlates of immune protection in this trial identified an association between the presence of antibody-dependent cellular cytotoxicity (ADCC) mediated by immunoglobulin G (IgG) antibodies (Abs) to HIV envelope (Env) V1/V2 loop structures and protection from infection, provided IgA Abs with competing specificity were not present. Systems serology analyses implicated a broader range of Ab-dependent functions in protection from HIV infection, including but not limited to ADCC and Ab-dependent NK cell activation (ADNKA) for secretion of IFN-γ and CCL4 and expression of the degranulation marker CD107a. The existence of such correlations in the absence of bNAbs in the RV144 trial suggest that NK cells could be instrumental in protecting against HIV infection by limiting viral spread through Fc-mediated functions such as ADCC and the production of antiviral cytokines/chemokines. Beside the engagement of FcγRIIIa or CD16 by the Fc portion of anti-Env IgG1 and IgG3 Abs, natural killer (NK) cells are also able to directly kill infected cells and produce cytokines/chemokines in an Ab-independent manner. Responsiveness of NK cells depends on the integration of activating and inhibitory signals through NK receptors, which is determined by a process during their development known as education. NK cell education requires the engagement of inhibitory NK receptors by their human leukocyte antigen ligands to establish tolerance to self while allowing NK cells to respond to self cells altered by virus infection, transformation, stress, and to allogeneic cells. Here, we review recent findings regarding the impact of inter-individual differences in NK cell education on Ab-dependent functions such as ADCC and ADNKA, including what is known about the HIV Env epitope specificity of ADCC competent Abs and the conformation of HIV Env on target cells used for ADCC assays.

## Introduction

There is great interest in developing an effective vaccine against HIV infection. It is generally acknowledged that inducing broadly neutralizing antibodies (bNAbs) would be a desirable goal for prophylactic HIV vaccines. The most potent bNAbs have been shown to protect against virus infection or to suppress viral replication in humanized mouse models and in rhesus macaques (1–6). Clinical trials conducted in HIV-infected humans, using the bNAbs VRC01 and 3BNC117, reduced HIV viral load by up to 2.5 logs (7, 8) and delayed viral rebound after antiretroviral therapy (ART) interruption (9, 10). However, there are still significant challenges to inducing such antibodies (Abs) through vaccination. BNAbs are rarely elicited in natural HIV infection and many exhibit high levels of affinity maturation (11–15). Despite this, progress has been made producing bNAbs in animal models using sequential cycles of boosting with defined immunogens (16). It is interesting to note that bNAbs able to protect humanized mice or rhesus macaques against challenge with HIV or simian/human immunodeficiency virus (SHIV) require an Fc region able to interact with Fc receptors (FcRs) on innate immune cells (17–19). One of these FcRs, FcγRIIIa, also known as CD16, is found on natural killer (NK) cells, macrophages, and monocyte subsets (20–22).

The HIV vaccine tested in the RV144 Thai trial is the only vaccine to date that conferred modest (approximately 31%) but significant protection against HIV infection (23). Protection was not associated with the presence of bNAbs or cytotoxic T cell responses (24). Rather, protection from HIV infection in trial participants was associated with the presence of anti-HIV envelope (Env) specific immunoglobulin G (IgG) non-neutralizing Abs (nNAbs) able to mediate Ab-dependent cellular cytotoxicity (ADCC) provided no potentially competitive IgA Abs were present (24–27). Follow-up analyses using systems serology approaches confirmed findings from correlation analyses and identified links between anti-Env V1/V2-specific IgG, IgG3, and IgG1, and Ab-dependent functions such as ADCC, Ab-dependent cellular phagocytosis, Ab-dependent complement deposition, and Ab-dependent NK cell activation (ADNKA) for secretion of IFN-γ and CCL4, and expression of CD107a in recipients of the RV144 vaccine (28). This raised the possibility that anti-HIV Env-specific nNAbs able to mediate ADCC and ADNKA activity may play a protective role against HIV infection.

Natural killer cells can be activated through Ab-dependent pathways that involve CD16 engagement by the Fc region of IgG1 and IgG3 Abs (29–35). They can also be activated by Ab-independent missing self recognition mechanisms based on how they were educated during development. Activating NK cells by either mechanism leads to secretion of chemokines and cytokines and to the release of cytotoxic granules that lyse target cells. ADNKA is the term used to describe the activation of NK cells for chemokine/cytokine secretion and degranulation by Ab-dependent stimuli. ADCC, on the other hand, denotes the lysis of target cells by NK cells in the presence of an Ab bridge. In the literature, these two activities have often been incorrectly referred to as ADCC. NK cells are important effector cells for these two Ab-dependent functions. Here, we will review recent findings on Ab-dependent functions mediated by NK cells and explore what is known regarding the influence of NK cell education on ADNKA and ADCC.

## NK Cell Education

Tolerance to self and the state of activation of NK cells is determined by an ontogenic process termed education. NK cell education requires the interaction of inhibitory NK receptors (iNKRs) with their cognate human leukocyte antigen (HLA) ligands on neighboring cells (36, 37). The education of NK cells determines how these cells will respond to infected, transformed, stressed, and allogeneic cells in an Ab-independent fashion. Education is a complex process whereby functionality is tuned by the number of iNKRs engaged, the strength of interactions between iNKRs and their ligands, and whether activating NK cell receptors are also engaged (38–44). NK cells lacking iNKRs for self-HLA ligands remain uneducated and hyporesponsive (45). iNKRs involved in NK cell education include NKG2A and the killer immunoglobulin-like receptors (KIR)3DL1, KIR2DL1, KIR2DL2, and KIR2DL3 (see Table 1). NKG2A is a C-type lectin receptor that forms a heterodimer with CD94 (46, 47). It interacts with non-classical major histocompatibility complex class I (MHC-I) HLA-E antigens presenting 9-mer peptides cleaved from the leader sequence of several MHC-I proteins (48, 49). Both NKG2A and HLA-E have limited sequence variability and their effects on NK cell education were initially reported to be similar from one person to another (50). The inhibitory KIRs (iKIRs) recognize subsets of HLA antigens together with peptides (51). KIR3DL1 interacts with a subset of HLA-A and -B antigens belonging to the HLA-Bw4 (Bw4) group (52–54). Bw4 antigens differ from the remaining HLA-Bw6 (Bw6) HLA-B variants at amino acids 77–83 of the HLA heavy chain (55). Bw6 isoforms do not interact with KIR3DL1 receptors such that KIR3DL1^+^ NK cells from individuals carrying no *Bw4* alleles are not educated through this receptor. KIR2DL3 and KIR2DL2 are encoded at the same locus and interact with HLA-C group 1 (C1) variants that have an asparagine at position 80 of the HLA heavy chain (56–58). The remaining HLA-C variants, belonging to the C2 group, have a lysine at this position and are ligands for KIR2DL1 (56). The KIR2DL3 receptor can also bind certain C2 variants, though with a lower affinity than either KIR2DL1 or KIR2DL2 (57, 59, 60). Therefore, KIR2DL3^+^ NK cells from individuals expressing a C1 ligand are educated, but remain uneducated or modestly educated through this receptor in individuals who are negative for C1 ligands. By contrast, KIR2DL1^+^ NK cells require the expression of a C2 ligand for education.

**Table 1:** Inhibitory natural killer (NK) cell receptors involved in NK cell education.

Receptor	Ligand	aa at position 80 of the human leukocyte antigen (HLA) heavy chain	Effect on education when ligand is present	Ligand levels in HIV-infected cells	Reference
NKG2A	HLA-E + leader peptide from HLA-A, -B, -C, and -G		Enhanced	Maintained	(48, 49)
Killer immunoglobulin-like receptors (KIR)3DL1	HLA-B*Bw4, HLA-A*23, *24, and *32	Isoleucine (*80I) or threonine (*80T)	Enhanced	Downmodulated	(52–54)
KIR2DL1	HLA-C2	Lysine	Enhanced	Maintained or downmodulated depending on HIV isolate	(56–60)
KIR2DL2	HLA-C1 (some HLA-C2)	Asparagine	Enhanced	Maintained or downmodulated depending on HIV isolate	(56–60)
KIR2DL3	HLA-C1 (some HLA-C2)	Asparagine	Enhanced	Maintained or downmodulated depending on HIV isolate	(56–60)

Genome-wide association studies (GWAS) confirm that genes influencing HIV viral load set point map to the *MHC-I* region on chromosome 6 (61, 62). MHC-I antigens encoded in this region form complexes with peptides, which are recognized by the T cell receptors on CD8^+^ T cells (63). It is well established that CD8^+^ T cells play an important role in HIV viral control (64–66). However, NKG2A and iKIR on NK cells also recognize MHC-I peptide complexes (48, 49, 52, 53, 56). Both epidemiological and functional studies have implicated iKIRs, particularly KIR3DL1, in combination with certain Bw4 variants in protection from HIV infection and slow disease progression in those already infected (67, 68). For example, individuals who are homozygous for KIR3DL1 *h/*y genotypes and co-carry HLA-B*57 (*h/*y + B*57) progress to AIDS more slowly and control HIV viral load better than *Bw6* hmz (67). KIR3DL1 *h/*y genotypes encode receptors expressed at high levels (69) while HLA-B*57 is a Bw4 variant that is also expressed on the cell surface at a high density and is a potent ligand for KIR3DL1 (44). The effect of this KIR/HLA combination on NK cell education is illustrated by the observation that KIR3DL1^+^ NK cells from *h/*y + B*57 carriers, compared to those from *Bw6* hmz, have a superior functional potential upon stimulation with HLA null cells and inhibit HIV replication more potently in autologous-infected CD4^+^ T cells through mechanisms that involve secretion of CC-chemokines (41, 70, 71). An upstream region of HLA-C that plays a role in determining HLA-C expression levels was also associated with HIV control in individuals of European American origin in GWAS studies (61, 62). While the mechanism underlying this association is related to HLA-C expression levels and the potency of CD8^+^ T cell recognition of HLA-C-HIV peptide complexes, the potential involvement of NK cells has not been excluded (72).

A dimorphism at position −21 in the leader peptide of HLA-B antigens influences the delivery of peptides to either an NKG2A or iKIR focused NK cell response (73). The amino acid at this position corresponds to the HLA leader peptide’s position 2, which is an anchor residue for HLA-E binding. A minority of HLA-B and all HLA-A and HLA-C antigens have a methionine at position −21 (−21M) of the leader sequence. −21M containing 9-mer peptides form stable complexes with HLA-E that are recognized by NKG2A. It is notable that the haplotypes carrying the −21M *HLA-B* alleles rarely encode Bw4 or C2 isoforms that are KIR3DL1 and KIR2DL1 ligands, respectively (73). By contrast, 9-mer peptides that have a threonine at the −21 (−21T) residue present in most HLA-B antigens, form poor complexes with HLA-E. Consequently, this −21M/T dimorphism defines two types of HLA haplotypes. One haplotype group, encoding −21M variants, is biased toward providing ligands for NKG2A and other group, encoding −21T variants, preferentially provides ligands for iKIR. This dimorphism appears to be clinically relevant in the context of HIV infection since the presence of −21M HLA-B antigens is associated with higher susceptibility to HIV infection in HIV-discordant couples and with poorer NK cell-mediated killing of HIV^+^ cells than are −21T HLA-B antigens (74, 75). Together, these findings prompt a reconsideration of epidemiological and NK cell functional studies in the light of the contribution of NKG2A versus iKIR responses to the activation of NK cell populations expressing defined patterns of iNKR.

## The Influence of NK Cell Education in ADNKA

Antibody-dependent NK cell activation measures NK cell activation following incubation with Ab opsonized targets cells. Even though ADNKA depends on the presence of Ab, NK cell education can also influence NK cell activation through ADNKA. Many of the earlier reports describing a role for NK cell education in ADNKA used the CEM.NKr.CCR5 (CEM) cell line coated with recombinant HIV Env gp120 as target cells (76). CEM cells express the CCR5 co-receptor for HIV entry and are resistant to direct NK cell killing (77–79). CEM cells are negative for Bw4 and C2 antigens but express C1 antigens (80).

A higher frequency of KIR3DL1^+^, than KIR3DL1^−^ NK cells, from carriers of *KIR3DL1/Bw4* genetic combinations secrete IFN-γ and express CD107a in responses to anti-HIV Ab opsonized gp120-coated CEM. This differential activation of KIR3DL1^+^ and KIR3DL1^−^ NK cell populations also occurs when the stimulus is HIV-infected or gp120-coated allogeneic primary CD4^+^ T cells (76). As well, a higher frequency of KIR2DL1^+^ than KIR2DL1^−^ NK cells from carriers of educating *KIR2DL1/HLA-C2* combinations secrete IFN-γ in response to HIV-infected autologous targets and gp120-coated CEM cells in the presence of anti-HIV Env-specific Abs in plasma from HIV^+^ individuals (81). By contrast, if NK cells are from carriers of the non-educating *KIR/HLA* pair *KIR2DL1/C1* hmz, KIR2DL1^+^ and KIR2DL1^−^ NK cells respond to anti-HIV Ab-dependent stimulation equivalently (81). These observations implicate NK cell education in NK cell responses to anti-HIV Ab opsonized gp120-coated CEM cells, infected allogeneic CEM cells, and gp120-coated primary CD4^+^ T cells. CD16 engagement is also important in ADNKA activity as NK cell activation is always higher in the presence versus absence anti-HIV-specific Abs.

Gooneratne et al. have speculated that ADCC activity directed at allogeneic HIV-infected cells may play a role in protecting against infection with allogeneic HIV-infected cells. Secretion of CCL4 from activated NK cells can bind the CCR5 HIV co-receptor and block HIV entry into new target cells (82). Activated NK cells also secrete cytotoxic granules that can lyse HIV-infected target cells (83). It is plausible that ADCC activity directed at allogeneic HIV-infected cells contributed to the modest protection conferred by the RV144 HIV vaccine trial, in which ADCC competent anti-Env-specific Abs were generated and to the protection conferred to infants who remain uninfected despite exposure to breast milk from HIV-infected mothers (24, 84).

There is a lack of consensus regarding whether educated NK cell populations respond more robustly than their uneducated counterparts to stimulation with anti-HIV opsonized autologous gp120-coated cells. KIR3DL1^+^ and KIR2DL1^+^ NK cells from carriers of *KIR/HLA* combinations able to support education through these receptors have been reported to respond better that their uneducated counterparts to HIV Ab-dependent activation (81, 85). These findings are consistent with results reported by Lang et al. (86). These observations have been interpreted as evidence that Ab-dependent activation of NK cells can overcome inhibitory signals mediated by the interaction of HLA ligand binding to self iKIR. However, this is not a general finding in that others have noted that ligands on autologous target cells to iNKR on educated NK cells suppress the activity of educated NK cells compared to that of their uneducated counterparts (87, 88). Further research is needed to understand what accounts for these discrepant results.

The experiments describing ADNKA in this section have used an inclusive gating strategy to compare how NK cell populations expressing, or not, one iNKR respond to anti-HIV opsonized target cells. When NK cells are stained inclusively for the presence of a single iNKR, the targeted population includes NK cells expressing other iNKRs not stained for. These other iNKRs could influence NK cell responses to HIV Ab opsonized target cells depending on which iNKR/HLA receptor ligand pairs contributed to the education of the NK cells studied. By using an Ab panel detecting KIR3DL1, KIR2DL1, KIR2DL3, and NKG2A on CD3^−^CD56^dim^ NK cells, it will be possible to focus on NK cell populations expressing one of these iNKR to the exclusion of the others. Such Ab panels that also detect multiple NK cell functions using Abs conjugated with different fluorochrome have been designed (89, 90). In future studies, these Ab panels should be used to exclusively gate on NK cell populations expressing single iNKRs that detect functions induced by anti-HIV Ab opsonized target cells. Such an experimental approach will allow for a more precise definition of NK cell responses within population expressing single educating receptors to activation through missing self recognition of the ligands for these iKIR on allogeneic CEM cells in addition to signals received *via* ligation of CD16 (91).

The frequency of NK cells responding to stimulation in ADNKA assays displays inter-individual variation. One possible mechanism underlying the range of NK cell effector responses in ADNKA assays is likely related to inter-individual differences in iNKR/HLA ligand effects on NK cell education. KIR3DL1 allotypes differ in their cell surface expression levels, with high, low, and null expression allotype groups (69, 92–95). These KIR3DL1 allotypes also differ in their affinity for particular HLA-B allotypes (44, 96). KIR2D receptors differ in their affinity for C1 and C2 antigens (57, 60). HLA-A, -B, and -C antigens also differ in their cell surface expression levels (44, 72, 97). Thus, these factors, the number of iNKR/HLA pairs participating in NK cell education in each study subject, and the presence of ligands on CEM cells that provide, or that fail to provide, inhibitory signals to NK cells may all influence NK cell activation levels in ADNKA assays. Several authors have tested expression levels for HLA-B and C allotypes and have examined the avidity of interactions of high and low expression KIR3DL1 receptor groups for HLA-B antigens with either an isoleucine or a threonine at position 80 of the HLA heavy chain (44, 69, 72, 98, 99). The putative influence of inter-personal immunogenetics on ADNKA activity could be explored by correlating ADNKA activation levels with KIR3DL1/HLA-B, KIR2DL1/HLA-C2, and KIR2DL3/HLA-C1 affinity and expression levels as has been described by Boudreau et al. (44). For ADNKA, activation through education-dependent missing self-recognition and CD16 signaling influence NK cell activation while for ADCC the effect of education-dependent missing self-recognition is minimized due to the low frequency of single positive (SP) iKIR+ NK cells positive for CD16. The comparison of assay results where one or more of these receptor ligand interactions is blocked may provide further insights into the role of signaling through iNKR or CD16 in ADNKA and ADCC.

## Measuring ADCC Activity

As opposed to ADNKA, ADCC measures target cell phenomena arising from the bridging of effector and target cells by an Ab whose Fc portion binds CD16 on effector cells and whose Fab portion recognizes an antigen on target cells. In the context of ADCC function-directed HIV Env gp120-coated target cells, the target antigens recognized by ADCC competent Abs are HIV Env (30, 78, 100). ADCC activity directed to HIV infected may also recognize Tat (100).

Early versions of anti-HIV ADCC assays measured ^51^Chromium release from target cells (101–103). These have been replaced by flow cytometry-based assays using either CEM cells coated with gp120 or gp140, HIV-infected CEM or HIV-infected primary CD4^+^ T cells as target cells. Primary HIV-infected target cells have included reactivated CD4^+^ T cells from HIV-infected subjects or CD4^+^ T cells infected with transmitted/founder (T/F) HIV isolates (104–106). The GranToxiLux ADCC (GTL-ADCC) assay measures the delivery of granzyme B (GzB) to target cells, an early step in the pathway leading to target cell lysis (83, 107, 108). In the GTL-ADCC assay, target cells are labeled with fluorescent and viability dyes before incubation with effector cells, either peripheral blood mononuclear cells (PBMCs) or NK cells in the presence of HIV-specific ADCC competent Abs and a GzB substrate. If ADCC is induced following incubation with HIV-specific Abs, effector cells will release GzB that will enter target cells and hydrolyze the GzB substrate, activating its fluorescence, which can be detected by flow cytometry. Thus, the GTL-ADCC assay provides an estimate of ADCC activity by measuring the number of viable targets that are positive for proteolytically active GzB.

Read outs for ADCC assays include the loss of target cells loaded with a fluorescent marker, infected with green fluorescent protein-tagged HIV, luciferase tagged HIV, or Gag p24^+^ cells (105, 106, 108–113). The lactate dehydrogenase (LDH) release ADCC assay measures the loss LDH from dying target cells by ELISA (76, 114). The widely used rapid and fluorometric ADCC has been shown to not measure ADCC but rather the uptake of the membrane dye PKH-26 used to label target cells by monocyte-mediated trogocytosis (115, 116).

## The Specificity of Anti-HIV ADCC Competent Abs

Both bNAbs and nNAbs can mediate ADCC activity provided they can stably bind to target cells (105, 106, 113, 117–121). HIV Env epitopes targeted by nNAbs include the immunodominant region of gp41 (122) and CD4-induced (CD4i) epitopes exposed by CD4 ligation of HIV Env on infected cells (111, 123, 124). Examples of prototypic anti-Env-specific Abs specific for a CD4i epitope is A32, which belongs to the anti-cluster A Ab group targeting the C1/C2 region and 17b, which recognizes the co-receptor binding site (CoRBS) (119, 125). Other nNAbs have been reported to recognize the CD4bs and the V3 loop of gp120, which are also targeted by bNAbs, though the nNAbs bind these epitopes in a manner that does not prevent HIV entry (126–130). At least some of the epitopes targeted by ADCC competent nNAbs are poorly exposed on CD4 unliganded cell surface Env trimers. This is mainly due to accessory proteins Nef and Vpu that downregulate cell surface CD4 making CD4i epitopes unavailable for Ab recognition (111, 120, 131, 132). Bruel et al. found that CEM cells infected with two laboratory-adapted HIV strains bound Abs from several classes of bNAb and nNAbs epitope specificity. If binding occurred, these Abs usually also mediated ADCC activity against these infected cells (106). However, when reactivated, HIV-infected cells from the reservoir of ART-treated HIV^+^ individuals or CD4^+^ T cells infected with T/F strains were used as target cells, several monoclonal nNAbs bound a lower frequency of infected cells with a lower affinity than did bNAbs. Furthermore, nNAbs, compared to bNAbs, exhibited poor ADCC activity against targets infected with such primary HIV strains (105, 106, 113, 117, 133). This phenomenon is likely related to the inability of nNAbs to access epitopes in the closed unliganded conformation of HIV Env (134).

Non-neutralizing Abs, particularly those specific for CD4i epitopes, preferentially bind HIV-uninfected bystander cells present in cultures with HIV^+^ CD4^+^ T cells (106, 135, 136). HIV-infected CD4^+^ T cells can shed HIV Env gp120 leaving behind gp41 stumps (136). The shed gp120 binds CD4 on the surface of uninfected bystander CD4^+^ T cells. This interaction has the potential to open the closed Env conformation exposing CD4i epitopes, making bystander enhanced targets for CD4i-specific ADCC competent Abs.

Strategies to improve the targeting of the open Env conformation by ADCC competent nNAbs has prompted exploring the use of CD4 mimetics to increase the susceptibility of HIV-infected cells to ADCC (106, 135, 137, 138). Richard et al. worked with CD4 mimetics that were unable to enhance the recognition of HIV-infected cells to A32 Abs by themselves (138). However, these small molecules initiated the opening of Env trimers enough to permit the binding of Abs such as 17b with specificity for a conserved epitope overlapping the CoRBS. Once 17b bound, the trimeric Env structure opened sufficiently to allow binding of A32 and susceptibility to ADCC activity (138).

It should be noted that most studies measuring anti-HIV ADCC activity have used gp120- or gp140-coated CEM cells as targets. While such targets are easy to prepare and convenient to use, the HIV Env on coated cells is monomeric and differs quantitatively and conformationally from trimeric Env found on the surface of HIV-infected cells. On coated cells, CD4 remains on the target cell surface while it is downregulated on infected cells unless Nef and/or Vpu HIV deletion mutants are used for infection. This needs to be kept in mind when interpreting the results of studies using coated cells as targets.

## The Influence of NK Cell Education on ADCC Activity

The GTL-ADCC assay using gp120-coated CEM cells as targets was used to show that education of effector populations through KIR3DL1 had no significant effect on the percent of GzB^+^ (%GzB^+^) target cells generated in a GTL-ADCC assay (139). There may be several explanations for this observation. One possibility is that NK cells are not the main effector cell in the GTL-ADCC assay. A drawback of using PBMCs as effector cells in ADCC assays is that it is difficult to draw conclusions regarding which effector population is responsible for GzB delivery to the target cells. Several Fcγ receptor-expressing cell types, including NK cells, monocytes/macrophages, and γδ T cells, are capable of mediating ADCC (107, 115, 140–144). To confirm that NK cells are the source of ADCC activity in the GTL-ADCC assay, Pollara et al. used effector PBMCs depleted of CD56^+^CD16^+^ NK cells and observed that ADCC responses declined by over 66% (145, 146). Purified NK cells and PBMCs from the same donors produced similar %GzB^+^ target cells (107). Together, these findings indicate that the GTL-ADCC assay is measuring NK cell-mediated ADCC responses.

**Figure 1:**
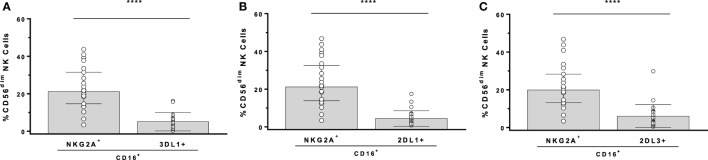
Comparison of the frequency of CD16^+^ cells among CD3^−^CD56^dim^ natural killer (NK) cells stained for antibodies with NKG2A, KIR3DL1 (3DL1^+^), KIR2DL1 (2DL1^+^), and KIR2DL3 (2DL3^+^). Comparison of single-positive (SP)NKG2A with SP3DL1 **(A)**, SPNKG2A with SP2DL1 **(B)**, and SPNKG2A with SP2DL3 **(C)**. Each point represents a single individual, bar height, and error bars represent median and interquartile range for the data set. Wilcoxon matched pairs tests were used to determine the significance of between group differences (****p < 0.0001).

In the GTL-ADCC assay, PBMC effector cells are a heterogeneous population that includes NK cells educated through 1, 2, or more iKIR and/or NKG2A. An Ab panel detecting KIR3DL1, KIR2DL1, KIR2DL3, and NKG2A on CD3^−^CD56^dim^ NK cells was used to gate exclusively on SPiNKR^+^ NK cells. NK cells SP for iKIR had significantly lower frequencies of CD16^+^ cells than did SPNKG2A^+^ or NKG2A^−^iKIR^−^ NK cells (147). iKIR^+^ NK cells are educated if they develop in a setting in which the iKIR’s ligand is co-expressed. The implication of this observation is that educated SPiKIR^+^ NK cells would be poor ADCC effector cells as a median of <5% of them are CD16^+^ (Figure 1). This could account for the lack of an effect of KIR3DL1-mediated NK cell education on the %GzB^+^ target cells generated in the GTL-ADCC assay (139, 147). Thus, it would be expected that NKG2A^+^ NK cells are superior to iKIR^+^NKG2A^−^ NK cells as effector cells in the GTL-ADCC assay. NKG2A/HLA-E interactions educate NKG2A^+^ NK cells and these receptor ligand pairs are widely expressed with limited inter-individual variation. Their influence on NK cell education would have limited between-subject variation. If ADCC activity is an important correlate of protection against HIV, these findings suggest that inter-individual variation in NK effector cell education based on which iKIR/HLA receptor/ligand pairs are present would have a minimal impact on ADCC potency at the level of HIV-infected target cell lysis or suppression of replication. Together, these findings illustrate that the potency of NK cell education and functional activation of NK effector cells does not predict the %GzB^+^ generated by ADCC.

In summary, factors important in determining ADNKA and ADCC activity differ from each other. The role of NK cell education in ADCC activity is limited by the low frequency of CD16^+^ NK cells among SPiKIR^+^ NK cells that have the potential to be educated through iKIR/HLA ligand interactions. Thus, a higher frequency of either uneducated NK cells or NK cells educated through NKG2A than those educated through iKIR are CD16^+^ and able to mediate ADCC. On the other hand, both CD16 engagement and missing self-recognition contribute to ADNKA. The consequences of these findings for HIV vaccines is that NK cell education should contribute minimally to inter-individual differences in target cell lysis by ADCC. Furthermore, NK cell activation by Ab-dependent HIV-infected cell stimuli will vary depending on how NK cells are educated, the nature of the stimulatory cell and effect of HIV infection on cell surface MHC-I expression (90, 148).

## Concluding Remarks

Arguing for a role for anti-HIV ADNKA and/or ADCC activity in protection from infection are the findings from the RV144 vaccine trial, which identified ADCC activity as a correlate of protection that was frequently linked to ADNKA activity (24, 27, 28). Moreover, antigenic drift from ADCC targeting Env epitopes has been documented, highlighting a role for ADCC being able to exert anti-HIV immune pressure (149). Of note, it is unlikely that bNAbs contributed to either of these findings as neither RV144 vaccinated individuals (24) nor most HIV^+^ persons make HIV-specific bNAbs. Suppression of HIV viral load in HIV-infected persons receiving the bNAb 3BNC117 is likely not solely due to virus neutralization as this treatment also appears to clear infected cells (133). Also, the beneficial effect of treatment with several bNAbs depends on IgG Fc region effects (17–19). On the other hand, several attempts to show that nNAbs can protect against infection in rhesus macaques infected with SHIV have failed, though passive transfer of these Abs may have suppressed viremia or restricted the number of T/F viruses in some cases (122, 150–152). By contrast, the passive transfer of the most active bNAbs mediates sterilizing protection in primate models (1–6). The protective role of anti-HIV nNAbs and/or how to manipulate the ability of these Abs to protect from HIV infection or how to use them therapeutically is an active area of research with several questions left to answer.

## Author Contributions

Substantial contributions to the conception or design of the work (NB, ZK, AT-M, CL, SK, and FD). Drafting the work or revising it critically for important intellectual content (NB, ZK, AT-M, CL, SK, and FD). Final approval of the version to be published (NB, ZK, AT-M, CL, SK, and FD). Agreement to be accountable for all aspects of the work in ensuring that questions related to the accuracy or integrity of any part of the work are appropriately investigated and resolved (NB, ZK, AT-M, CL, SK, and FD).

## Conflict of Interest Statement

The authors declare that the research was conducted in the absence of any commercial or financial relationships that could be construed as a potential conflict of interest.

## References

[1] Klein F Halper-Stromberg A Horwitz JA Gruell H Scheid JF Bournazos S HIV therapy by a combination of broadly neutralizing antibodies in humanized mice. Nature (2012) 492(7427):118–22. 10.1038/nature11604 23103874 PMC3809838

[2] Horwitz JA Halper-Stromberg A Mouquet H Gitlin AD Tretiakova A Eisenreich TR HIV-1 suppression and durable control by combining single broadly neutralizing antibodies and antiretroviral drugs in humanized mice. Proc Natl Acad Sci U S A (2013) 110(41):16538–43. 10.1073/pnas.1315295110 24043801 PMC3799352

[3] Barouch DH Whitney JB Moldt B Klein F Oliveira TY Liu J Therapeutic efficacy of potent neutralizing HIV-1-specific monoclonal antibodies in SHIV-infected rhesus monkeys. Nature (2013) 503(7475):224–8. 10.1038/nature12744 24172905 PMC4017780

[4] Shingai M Nishimura Y Klein F Mouquet H Donau OK Plishka R Antibody-mediated immunotherapy of macaques chronically infected with SHIV suppresses viraemia. Nature (2013) 503(7475):277–80. 10.1038/nature12746 24172896 PMC4133787

[5] Gautam R Nishimura Y Pegu A Nason MC Klein F Gazumyan A A single injection of anti-HIV-1 antibodies protects against repeated SHIV challenges. Nature (2016) 533(7601):105–9. 10.1038/nature17677 27120156 PMC5127204

[6] Moldt B Rakasz EG Schultz N Chan-Hui PY Swiderek K Weisgrau KL Highly potent HIV-specific antibody neutralization in vitro translates into effective protection against mucosal SHIV challenge in vivo. Proc Natl Acad Sci U S A (2012) 109(46):18921–5. 10.1073/pnas.1214785109 23100539 PMC3503218

[7] Caskey M Klein F Lorenzi JC Seaman MS West AP Jr Buckley N Viraemia suppressed in HIV-1-infected humans by broadly neutralizing antibody 3BNC117. Nature (2015) 522(7557):487–91. 10.1038/nature14411 25855300 PMC4890714

[8] Lynch RM Boritz E Coates EE DeZure A Madden P Costner P Virologic effects of broadly neutralizing antibody VRC01 administration during chronic HIV-1 infection. Sci Transl Med (2015) 7(319):319ra206. 10.1126/scitranslmed.aad5752 26702094 PMC12366723

[9] Scheid JF Horwitz JA Bar-On Y Kreider EF Lu CL Lorenzi JC HIV-1 antibody 3BNC117 suppresses viral rebound in humans during treatment interruption. Nature (2016) 535(7613):556–60. 10.1038/nature18929 27338952 PMC5034582

[10] Bar KJ Sneller MC Harrison LJ Justement JS Overton ET Petrone ME Effect of HIV antibody VRC01 on viral rebound after treatment interruption. N Engl J Med (2016) 375(21):2037–50. 10.1056/NEJMoa1608243 27959728 PMC5292134

[11] Burton DR Hangartner L. Broadly neutralizing antibodies to HIV and their role in vaccine design. Annu Rev Immunol (2016) 34:635–59. 10.1146/annurev-immunol-041015-055515 27168247 PMC6034635

[12] West AP Jr Scharf L Scheid JF Klein F Bjorkman PJ Nussenzweig MC. Structural insights on the role of antibodies in HIV-1 vaccine and therapy. Cell (2014) 156(4):633–48. 10.1016/j.cell.2014.01.052 24529371 PMC4041625

[13] Rusert P Kouyos RD Kadelka C Ebner H Schanz M Huber M Determinants of HIV-1 broadly neutralizing antibody induction. Nat Med (2016) 22(11):1260–7. 10.1038/nm.4187 27668936

[14] Mouquet H Antibody B cell responses in HIV-1 infection. Trends Immunol (2014) 35(11):549–61. 10.1016/j.it.2014.08.007 25240985

[15] Klein F Diskin R Scheid JF Gaebler C Mouquet H Georgiev IS Somatic mutations of the immunoglobulin framework are generally required for broad and potent HIV-1 neutralization. Cell (2013) 153(1):126–38. 10.1016/j.cell.2013.03.018 23540694 PMC3792590

[16] Briney B Sok D Jardine JG Kulp DW Skog P Menis S Tailored immunogens direct affinity maturation toward HIV neutralizing antibodies. Cell (2016) 166(6):1459–70.e11. 10.1016/j.cell.2016.08.005 27610570 PMC5018249

[17] Hessell AJ Hangartner L Hunter M Havenith CE Beurskens FJ Bakker JM Fc receptor but not complement binding is important in antibody protection against HIV. Nature (2007) 449(7158):101–4. 10.1038/nature06106 17805298

[18] Bournazos S Klein F Pietzsch J Seaman MS Nussenzweig MC Ravetch JV. Broadly neutralizing anti-HIV-1 antibodies require Fc effector functions for in vivo activity. Cell (2014) 158(6):1243–53. 10.1016/j.cell.2014.08.023 25215485 PMC4167398

[19] Halper-Stromberg A Lu CL Klein F Horwitz JA Bournazos S Nogueira L Broadly neutralizing antibodies and viral inducers decrease rebound from HIV-1 latent reservoirs in humanized mice. Cell (2014) 158(5):989–99. 10.1016/j.cell.2014.07.043 25131989 PMC4163911

[20] Nimmerjahn F Ravetch JV. Fcgamma receptors as regulators of immune responses. Nat Rev Immunol (2008) 8(1):34–47. 10.1038/nri2206 18064051

[21] Trinchieri G Valiante N. Receptors for the Fc fragment of IgG on natural killer cells. Nat Immun (1993) 12(4–5):218–34. 8257828

[22] Bournazos S Ravetch JV Fcgamma receptor pathways during active and passive immunization. Immunol Rev (2015) 268(1):88–103. 10.1111/imr.12343 26497515 PMC7556827

[23] Rerks-Ngarm S Pitisuttithum P Nitayaphan S Kaewkungwal J Chiu J Paris R Vaccination with ALVAC and AIDSVAX to prevent HIV-1 infection in Thailand. N Engl J Med (2009) 361(23):2209–20. 10.1056/NEJMoa0908492 19843557

[24] Haynes BF Gilbert PB McElrath MJ Zolla-Pazner S Tomaras GD Alam SM Immune-correlates analysis of an HIV-1 vaccine efficacy trial. N Engl J Med (2012) 366(14):1275–86. 10.1056/NEJMoa1113425 22475592 PMC3371689

[25] Yates NL Liao HX Fong Y DeCamp A Vandergrift NA Williams WT Vaccine-induced Env V1-V2 IgG3 correlates with lower HIV-1 infection risk and declines soon after vaccination. Sci Transl Med (2014) 6(228):228ra39. 10.1126/scitranslmed.3007730 24648342 PMC4116665

[26] Tomaras GD Ferrari G Shen X Alam SM Liao HX Pollara J Vaccine-induced plasma IgA specific for the C1 region of the HIV-1 envelope blocks binding and effector function of IgG. Proc Natl Acad Sci U S A (2013) 110:9019–24. 10.1073/pnas.1301456110 23661056 PMC3670311

[27] Chung AW Ghebremichael M Robinson H Brown E Choi I Lane S Polyfunctional Fc-effector profiles mediated by IgG subclass selection distinguish RV144 and VAX003 vaccines. Sci Transl Med (2014) 6(228):228ra38. 10.1126/scitranslmed.3007736 24648341

[28] Chung AW Kumar MP Arnold KB Yu WH Schoen MK Dunphy LJ Dissecting polyclonal vaccine-induced humoral immunity against HIV using systems serology. Cell (2015) 163(4):988–98. 10.1016/j.cell.2015.10.027 26544943 PMC5490491

[29] Brier AM Chess L Schlossman SF. Human antibody-dependent cellular cytotoxicity. Isolation and identification of a subpopulation of peripheral blood lymphocytes which kill antibody-coated autologous target cells. J Clin Invest (1975) 56(6):1580–6. 10.1172/JCI108240 53242 PMC333137

[30] Koup RA Sullivan JL Levine PH Brewster F Mahr A Mazzara G Antigenic specificity of antibody-dependent cell-mediated cytotoxicity directed against human immunodeficiency virus in antibody-positive sera. J Virol (1989) 63(2):584–90. 2536094 10.1128/jvi.63.2.584-590.1989 PMC247727

[31] Bruggemann M Williams GT Bindon CI Clark MR Walker MR Jefferis R Comparison of the effector functions of human immunoglobulins using a matched set of chimeric antibodies. J Exp Med (1987) 166(5):1351–61. 10.1084/jem.166.5.1351 3500259 PMC2189658

[32] Warncke M Calzascia T Coulot M Balke N Touil R Kolbinger F Different adaptations of IgG effector function in human and nonhuman primates and implications for therapeutic antibody treatment. J Immunol (2012) 188(9):4405–11. 10.4049/jimmunol.1200090 22461693

[33] Boesch AW Brown EP Cheng HD Ofori MO Normandin E Nigrovic PA Highly parallel characterization of IgG Fc binding interactions. MAbs (2014) 6(4):915–27. 10.4161/mabs.28808 24927273 PMC4171026

[34] Fauriat C Long EO Ljunggren HG Bryceson YT. Regulation of human NK-cell cytokine and chemokine production by target cell recognition. Blood (2010) 115(11):2167–76. 10.1182/blood-2009-08-238469 19965656 PMC2844017

[35] Bruhns P Iannascoli B England P Mancardi DA Fernandez N Jorieux S Specificity and affinity of human Fcgamma receptors and their polymorphic variants for human IgG subclasses. Blood (2009) 113(16):3716–25. 10.1182/blood-2008-09-179754 19018092

[36] Kim S Poursine-Laurent J Truscott SM Lybarger L Song YJ Yang L Licensing of natural killer cells by host major histocompatibility complex class I molecules. Nature (2005) 436(7051):709–13. 10.1038/nature03847 16079848

[37] Anfossi N Andre P Guia S Falk CS Roetynck S Stewart CA Human NK cell education by inhibitory receptors for MHC class I. Immunity (2006) 25(2):331–42. 10.1016/j.immuni.2006.06.013 16901727

[38] Brodin P Lakshmikanth T Johansson S Karre K Hoglund P. The strength of inhibitory input during education quantitatively tunes the functional responsiveness of individual natural killer cells. Blood (2009) 113(11):2434–41. 10.1182/blood-2008-05-156836 18974374

[39] Fauriat C Ivarsson MA Ljunggren HG Malmberg KJ Michaelsson J. Education of human natural killer cells by activating killer cell immunoglobulin-like receptors. Blood (2010) 115(6):1166–74. 10.1182/blood-2009-09-245746 19903900

[40] Johansson S Johansson M Rosmaraki E Vahlne G Mehr R Salmon-Divon M Natural killer cell education in mice with single or multiple major histocompatibility complex class I molecules. J Exp Med (2005) 201(7):1145–55. 10.1084/jem.20050167 15809355 PMC2213126

[41] Boulet S Song R Kamya P Bruneau J Shoukry NH Tsoukas CM HIV protective KIR3DL1 and HLA-B genotypes influence NK cell function following stimulation with HLA-devoid cells. J Immunol (2010) 184(4):2057–64. 10.4049/jimmunol.0902621 20061407

[42] Kamya P Tsoukas CM Boulet S Routy JP Thomas R Cote P T cell activation does not drive CD4 decline in longitudinally followed HIV-infected elite controllers. AIDS Res Ther (2011) 8(1):20. 10.1186/1742-6405-8-20 21679427 PMC3131237

[43] Kim S Sunwoo JB Yang L Choi T Song YJ French AR HLA alleles determine differences in human natural killer cell responsiveness and potency. Proc Natl Acad Sci U S A (2008) 105(8):3053–8. 10.1073/pnas.0712229105 18287063 PMC2268583

[44] Boudreau JE Mulrooney TJ Le Luduec JB Barker E Hsu KC. KIR3DL1 and HLA-B density and binding calibrate NK education and response to HIV. J Immunol (2016) 196(8):3398–410. 10.4049/jimmunol.1502469 26962229 PMC4868784

[45] Fernandez NC Treiner E Vance RE Jamieson AM Lemieux S Raulet DH. A subset of natural killer cells achieves self-tolerance without expressing inhibitory receptors specific for self-MHC molecules. Blood (2005) 105(11):4416–23. 10.1182/blood-2004-08-3156 15728129 PMC1895026

[46] Brooks AG Posch PE Scorzelli CJ Borrego F Coligan JE. NKG2A complexed with CD94 defines a novel inhibitory natural killer cell receptor. J Exp Med (1997) 185(4):795–800. 10.1084/jem.185.4.795 9034158 PMC2196137

[47] Lazetic S Chang C Houchins JP Lanier LL Phillips JH. Human natural killer cell receptors involved in MHC class I recognition are disulfide-linked heterodimers of CD94 and NKG2 subunits. J Immunol (1996) 157(11):4741–5. 8943374

[48] Braud VM Allan DS O’Callaghan CA Soderstrom K D’Andrea A Ogg GS HLA-E binds to natural killer cell receptors CD94/NKG2A, B and C. Nature (1998) 391(6669):795–9. 10.1038/35869 9486650

[49] Braud V Jones EY McMichael A. The human major histocompatibility complex class Ib molecule HLA-E binds signal sequence-derived peptides with primary anchor residues at positions 2 and 9. Eur J Immunol (1997) 27(5):1164–9. 10.1002/eji.1830270517 9174606

[50] Yawata M Yawata N Draghi M Partheniou F Little AM Parham P MHC class I-specific inhibitory receptors and their ligands structure diverse human NK-cell repertoires toward a balance of missing self-response. Blood (2008) 112(6):2369–80. 10.1182/blood-2008-03-143727 18583565 PMC2532809

[51] Saunders PM Vivian JP O’Connor GM Sullivan LC Pymm P Rossjohn J A bird’s eye view of NK cell receptor interactions with their MHC class I ligands. Immunol Rev (2015) 267(1):148–66. 10.1111/imr.12319 26284476

[52] Cella M Longo A Ferrara GB Strominger JL Colonna M. NK3-specific natural killer cells are selectively inhibited by Bw4-positive HLA alleles with isoleucine 80. J Exp Med (1994) 180(4):1235–42. 10.1084/jem.180.4.1235 7931060 PMC2191670

[53] Gumperz JE Litwin V Phillips JH Lanier LL Parham P. The Bw4 public epitope of HLA-B molecules confers reactivity with natural killer cell clones that express NKB1, a putative HLA receptor. J Exp Med (1995) 181(3):1133–44. 10.1084/jem.181.3.1133 7532677 PMC2191933

[54] Stern M Ruggeri L Capanni M Mancusi A Velardi A Human leukocyte antigens A23, A24, and A32 but not A25 are ligands for KIR3DL1. Blood (2008) 112(3):708–10. 10.1182/blood-2008-02-137521 18502829

[55] Wan AM Ennis P Parham P Holmes N. The primary structure of HLA-A32 suggests a region involved in formation of the Bw4/Bw6 epitopes. J Immunol (1986) 137(11):3671–4. 2431040

[56] Colonna M Borsellino G Falco M Ferrara GB Strominger JL. HLA-C is the inhibitory ligand that determines dominant resistance to lysis by NK1- and NK2-specific natural killer cells. Proc Natl Acad Sci U S A (1993) 90(24):12000–4. 10.1073/pnas.90.24.12000 8265660 PMC48113

[57] Moesta AK Norman PJ Yawata M Yawata N Gleimer M Parham P. Synergistic polymorphism at two positions distal to the ligand-binding site makes KIR2DL2 a stronger receptor for HLA-C than KIR2DL3. J Immunol (2008) 180(6):3969–79. 10.4049/jimmunol.180.6.3969 18322206

[58] Winter CC Gumperz JE Parham P Long EO Wagtmann N. Direct binding and functional transfer of NK cell inhibitory receptors reveal novel patterns of HLA-C allotype recognition. J Immunol (1998) 161(2):571–7. 9670929

[59] Moesta AK Parham P. Diverse functionality among human NK cell receptors for the C1 epitope of HLA-C: KIR2DS2, KIR2DL2, and KIR2DL3. Front Immunol (2012) 3:336. 10.3389/fimmu.2012.00336 23189078 PMC3504360

[60] Hilton HG Vago L Older Aguilar AM Moesta AK Graef T Abi-Rached L Mutation at positively selected positions in the binding site for HLA-C shows that KIR2DL1 is a more refined but less adaptable NK cell receptor than KIR2DL3. J Immunol (2012) 189(3):1418–30. 10.4049/jimmunol.1100431 22772445 PMC3439511

[61] Fellay J Shianna KV Ge D Colombo S Ledergerber B Weale M A whole-genome association study of major determinants for host control of HIV-1. Science (2007) 317(5840):944–7. 10.1126/science.1143767 17641165 PMC1991296

[62] Pereyra F Jia X McLaren PJ Telenti A de Bakker PI Walker BD The major genetic determinants of HIV-1 control affect HLA class I peptide presentation. Science (2010) 330(6010):1551–7. 10.1126/science.1195271 21051598 PMC3235490

[63] Davis MM Boniface JJ Reich Z Lyons D Hampl J Arden B Ligand recognition by alpha beta T cell receptors. Annu Rev Immunol (1998) 16:523–44. 10.1146/annurev.immunol.16.1.523 9597140

[64] Walker CM Moody DJ Stites DP Levy JA. CD8+ lymphocytes can control HIV infection in vitro by suppressing virus replication. Science (1986) 234(4783):1563–6. 10.1126/science.2431484 2431484

[65] Koup RA Safrit JT Cao Y Andrews CA McLeod G Borkowsky W Temporal association of cellular immune responses with the initial control of viremia in primary human immunodeficiency virus type 1 syndrome. J Virol (1994) 68(7):4650–5. 8207839 10.1128/jvi.68.7.4650-4655.1994 PMC236393

[66] McMichael AJ Rowland-Jones SL Cellular immune responses to HIV. Nature (2001) 410(6831):980–7. 10.1038/35073658 11309628

[67] Martin MP Qi Y Gao X Yamada E Martin JN Pereyra F Innate partnership of HLA-B and KIR3DL1 subtypes against HIV-1. Nat Genet (2007) 39(6):733–40. 10.1038/ng2035 17496894 PMC4135476

[68] Boulet S Kleyman M Kim JY Kamya P Sharafi S Simic N A combined genotype of KIR3DL1 high expressing alleles and HLA-B*57 is associated with a reduced risk of HIV infection. AIDS (2008) 22(12):1487–91. 10.1097/QAD.0b013e3282ffde7e 18614872

[69] Yawata M Yawata N Draghi M Little AM Partheniou F Parham P. Roles for HLA and KIR polymorphisms in natural killer cell repertoire selection and modulation of effector function. J Exp Med (2006) 203(3):633–45. 10.1084/jem.20051884 16533882 PMC2118260

[70] Kamya P Boulet S Tsoukas CM Routy JP Thomas R Cote P Receptor-ligand requirements for increased NK cell poly-functional potential in *h/*y+B57 HIV-1 infected slow progressors. J Virol (2011) 85(12):5949–60. 10.1128/JVI.02652-10 21471235 PMC3126301

[71] Song R Lisovsky I Lebouche B Routy JP Bruneau J Bernard NF. HIV protective KIR3DL1/S1-HLA-B genotypes influence NK cell-mediated inhibition of HIV replication in autologous CD4 targets. PLoS Pathog (2014) 10(1):e1003867. 10.1371/journal.ppat.1003867 24453969 PMC3894215

[72] Apps R Qi Y Carlson JM Chen H Gao X Thomas R Influence of HLA-C expression level on HIV control. Science (2013) 340(6128):87–91. 10.1126/science.1232685 23559252 PMC3784322

[73] Horowitz A Djaoud Z Nemat-Gorgani N Blokhuis J Hilton HG Beziat V Class I HLA haplotypes form two schools that educate NK cells in different ways. Sci Immunol (2016) 1(3):1–14. 10.1126/sciimmunol.aag1672 PMC5110269 27868107

[74] Merino AM Sabbaj S Easlick J Goepfert P Kaslow RA Tang J. Dimorphic HLA-B signal peptides differentially influence HLA-E- and natural killer cell-mediated cytolysis of HIV-1-infected target cells. Clin Exp Immunol (2013) 174(3):414–23. 10.1111/cei.12187 23952339 PMC3826307

[75] Merino AM Song W He D Mulenga J Allen S Hunter E HLA-B signal peptide polymorphism influences the rate of HIV-1 acquisition but not viral load. J Infect Dis (2012) 205(12):1797–805. 10.1093/infdis/jis275 22492862 PMC3571229

[76] Gooneratne SL Richard J Lee WS Finzi A Kent SJ Parsons MS. Slaying the Trojan horse: natural killer cells exhibit robust anti-HIV-1 antibody-dependent activation and cytolysis against allogeneic T cells. J Virol (2015) 89(1):97–109. 10.1128/JVI.02461-14 25320293 PMC4301139

[77] Howell DN Andreotti PE Dawson JR Cresswell P. Natural killing target antigens as inducers of interferon: studies with an immunoselected, natural killing-resistant human T lymphoblastoid cell line. J Immunol (1985) 134(2):971–6. 3871222

[78] Lyerly HK Reed DL Matthews TJ Langlois AJ Ahearne PA Petteway SR Jr Anti-GP 120 antibodies from HIV seropositive individuals mediate broadly reactive anti-HIV ADCC. AIDS Res Hum Retroviruses (1987) 3(4):409–22. 10.1089/aid.1987.3.409 2833917

[79] Trkola A Matthews J Gordon C Ketas T Moore JP. A cell line-based neutralization assay for primary human immunodeficiency virus type 1 isolates that use either the CCR5 or the CXCR4 coreceptor. J Virol (1999) 73(11):8966–74. 10516002 10.1128/jvi.73.11.8966-8974.1999 PMC112928

[80] Lisovsky I Isitman G Bruneau J Bernard NF. Functional analysis of NK cell subsets activated by 721.221 and K562 HLA-null cells. J Leukoc Biol (2015) 97(4):761–7. 10.1189/jlb.4AB1014-499R 25713086

[81] Gooneratne SL Center RJ Kent SJ Parsons MS. Functional advantage of educated KIR2DL1(+) natural killer cells for anti-HIV-1 antibody-dependent activation. Clin Exp Immunol (2016) 184(1):101–9. 10.1111/cei.12752 26647083 PMC4778096

[82] Oliva A Kinter AL Vaccarezza M Rubbert A Catanzaro A Moir S Natural killer cells from human immunodeficiency virus (HIV)-infected individuals are an important source of CC-chemokines and suppress HIV-1 entry and replication in vitro. J Clin Invest (1998) 102(1):223–31. 10.1172/JCI2323 9649576 PMC509084

[83] Packard BZ Telford WG Komoriya A Henkart PA. Granzyme B activity in target cells detects attack by cytotoxic lymphocytes. J Immunol (2007) 179(6):3812–20. 10.4049/jimmunol.179.6.3812 17785818

[84] Mabuka J Nduati R Odem-Davis K Peterson D Overbaugh J. HIV-specific antibodies capable of ADCC are common in breastmilk and are associated with reduced risk of transmission in women with high viral loads. PLoS Pathog (2012) 8(6):e1002739. 10.1371/journal.ppat.1002739 22719248 PMC3375288

[85] Parsons MS Wren L Isitman G Navis M Stratov I Bernard NF HIV infection abrogates the functional advantage of natural killer cells educated through KIR3DL1/HLA-Bw4 interactions to mediate anti-HIV antibody-dependent cellular cytotoxicity. J Virol (2012) 86(8):4488–95. 10.1128/JVI.06112-11 22345455 PMC3318670

[86] Lang P Pfeiffer M Handgretinger R Schumm M Demirdelen B Stanojevic S Clinical scale isolation of T cell-depleted CD56+ donor lymphocytes in children. Bone Marrow Transplant (2002) 29(6):497–502. 10.1038/sj.bmt.1703406 11960269

[87] Ward JP Bonaparte MI Barker E. HLA-C and HLA-E reduce antibody-dependent natural killer cell-mediated cytotoxicity of HIV-infected primary T cell blasts. AIDS (2004) 18(13):1769–79. 10.1097/00002030-200409030-00005 15316337

[88] Tarek N Le Luduec JB Gallagher MM Zheng J Venstrom JM Chamberlain E Unlicensed NK cells target neuroblastoma following anti-GD2 antibody treatment. J Clin Invest (2012) 122(9):3260–70. 10.1172/JCI62749 22863621 PMC3428088

[89] Lisovsky I Isitman G Song R DaFonseca S Tremblay-McLean A Lebouche B A higher frequency of NKG2A+ than of NKG2A− NK cells respond to autologous HIV-infected CD4 cells irrespective of whether they co-express KIR3DL1. J Virol (2015) 89(19):9909–19. 10.1128/JVI.01546-15 26202228 PMC4577891

[90] Lisovsky I Isitman G Tremblay-McLean A Song R DaFonseca S Lebouche B The differential impact of natural killer (NK) cell education via KIR2DL3 and KIR3DL1 on CCL4 secretion in the context of in-vitro HIV infection. Clin Exp Immunol (2016) 186(3):336–46. 10.1111/cei.12849 27506421 PMC5108064

[91] Ljunggren HG Karre K. In search of the ‘missing self’: MHC molecules and NK cell recognition. Immunol Today (1990) 11(7):237–44. 10.1016/0167-5699(90)90097-S 2201309

[92] Ahlenstiel G Martin MP Gao X Carrington M Rehermann B. Distinct KIR/HLA compound genotypes affect the kinetics of human antiviral natural killer cell responses. J Clin Invest (2008) 118(3):1017–26. 10.1172/JCI32400 18246204 PMC2214845

[93] Trundley A Frebel H Jones D Chang C Trowsdale J. Allelic expression patterns of KIR3DS1 and 3DL1 using the Z27 and DX9 antibodies. Eur J Immunol (2007) 37(3):780–7. 10.1002/eji.200636773 17301953

[94] Gardiner CM Guethlein LA Shilling HG Pando M Carr WH Rajalingam R Different NK cell surface phenotypes defined by the DX9 antibody are due to KIR3DL1 gene polymorphism. J Immunol (2001) 166(5):2992–3001. 10.4049/jimmunol.166.5.2992 11207248

[95] Boudreau JE Le Luduec JB Hsu KC. Development of a novel multiplex PCR assay to detect functional subtypes of KIR3DL1 alleles. PLoS One (2014) 9(6):e99543. 10.1371/journal.pone.0099543 24919192 PMC4053526

[96] Hilton HG Moesta AK Guethlein LA Blokhuis J Parham P Norman PJ. The production of KIR-Fc fusion proteins and their use in a multiplex HLA class I binding assay. J Immunol Methods (2015) 425:79–87. 10.1016/j.jim.2015.06.012 26096968 PMC4604020

[97] Ramsuran V Kulkarni S O’Huigin C Yuki Y Augusto DG Gao X Epigenetic regulation of differential HLA-A allelic expression levels. Hum Mol Genet (2015) 24(15):4268–75. 10.1093/hmg/ddv158 25935001 PMC4492392

[98] Carr WH Pando MJ Parham P. KIR3DL1 polymorphisms that affect NK cell inhibition by HLA-Bw4 ligand. J Immunol (2005) 175(8):5222–9. 10.4049/jimmunol.175.8.5222 16210627

[99] O’Connor GM Guinan KJ Cunningham RT Middleton D Parham P Gardiner CM. Functional polymorphism of the KIR3DL1/S1 receptor on human NK cells. J Immunol (2007) 178(1):235–41. 10.4049/jimmunol.178.1.235 17182560

[100] Kulkarni A Kurle S Shete A Ghate M Godbole S Madhavi V Indian long-term non-progressors show broad ADCC responses with preferential recognition of V3 region of envelope and a region from Tat protein. Front Immunol (2017) 8:5. 10.3389/fimmu.2017.00005 28154562 PMC5243827

[101] Baum LL Cassutt KJ Knigge K Khattri R Margolick J Rinaldo C HIV-1 gp120-specific antibody-dependent cell-mediated cytotoxicity correlates with rate of disease progression. J Immunol (1996) 157(5):2168–73. 8757343

[102] Ahmad A Morisset R Thomas R Menezes J. Evidence for a defect of antibody-dependent cellular cytotoxic (ADCC) effector function and anti-HIV gp120/41-specific ADCC-mediating antibody titres in HIV-infected individuals. J Acquir Immune Defic Syndr (1994) 7(5):428–37. 7908983

[103] Tyler DS Stanley SD Nastala CA Austin AA Bartlett JA Stine KC Alterations in antibody-dependent cellular cytotoxicity during the course of HIV-1 infection. Humoral and cellular defects. J Immunol (1990) 144(9):3375–84. 2329275

[104] Kramski M Parsons MS Stratov I Kent SJ. HIV-specific antibody immunity mediated through NK cells and monocytes. Curr HIV Res (2013) 11(5):388–406. 10.2174/1570162X113116660061 24191935

[105] Bruel T Guivel-Benhassine F Amraoui S Malbec M Richard L Bourdic K Elimination of HIV-1-infected cells by broadly neutralizing antibodies. Nat Commun (2016) 7:10844. 10.1038/ncomms10844 26936020 PMC4782064

[106] Bruel T Guivel-Benhassine F Lorin V Lortat-Jacob H Baleux F Bourdic K Lack of ADCC breadth of human nonneutralizing anti-HIV-1 antibodies. J Virol (2017) 91(8):e2440–16. 10.1128/JVI.02440-16 28122982 PMC5375671

[107] Pollara J Hart L Brewer F Pickeral J Packard BZ Hoxie JA High-throughput quantitative analysis of HIV-1 and SIV-specific ADCC-mediating antibody responses. Cytometry A (2011) 79(8):603–12. 10.1002/cyto.a.21084 21735545 PMC3692008

[108] Smalls-Mantey A Doria-Rose N Klein R Patamawenu A Migueles SA Ko SY Antibody-dependent cellular cytotoxicity against primary HIV-infected CD4+ T cells is directly associated with the magnitude of surface IgG binding. J Virol (2012) 86(16):8672–80. 10.1128/JVI.00287-12 22674985 PMC3421757

[109] Alpert MD Harvey JD Lauer WA Reeves RK Piatak M Jr Carville A ADCC develops over time during persistent infection with live-attenuated SIV and is associated with complete protection against SIV(mac)251 challenge. PLoS Pathog (2012) 8(8):e1002890. 10.1371/journal.ppat.1002890 22927823 PMC3426556

[110] Pham TN Lukhele S Hajjar F Routy JP Cohen EA HIV Nef and Vpu protect HIV-infected CD4+ T cells from antibody-mediated cell lysis through down-modulation of CD4 and BST2. Retrovirology (2014) 11:15 10.1186/1742-4690-11-15 24498878 PMC3930549

[111] Veillette M Desormeaux A Medjahed H Gharsallah NE Coutu M Baalwa J Interaction with cellular CD4 exposes HIV-1 envelope epitopes targeted by antibody-dependent cell-mediated cytotoxicity. J Virol (2014) 88(5):2633–44. 10.1128/JVI.03230-13 24352444 PMC3958102

[112] Richard J Veillette M Batraville LA Coutu M Chapleau JP Bonsignori M Flow cytometry-based assay to study HIV-1 gp120 specific antibody-dependent cellular cytotoxicity responses. J Virol Methods (2014) 208:107–14. 10.1016/j.jviromet.2014.08.003 25125129

[113] von Bredow B Arias JF Heyer LN Moldt B Le K Robinson JE Comparison of antibody-dependent cell-mediated cytotoxicity and virus neutralization by HIV-1 Env-specific monoclonal antibodies. J Virol (2016) 90(13):6127–39. 10.1128/JVI.00347-16 27122574 PMC4907221

[114] Korzeniewski C Callewaert DM. An enzyme-release assay for natural cytotoxicity. J Immunol Methods (1983) 64(3):313–20. 10.1016/0022-1759(83)90438-6 6199426

[115] Kramski M Schorcht A Johnston AP Lichtfuss GF Jegaskanda S De Rose R Role of monocytes in mediating HIV-specific antibody-dependent cellular cytotoxicity. J Immunol Methods (2012) 384(1–2):51–61. 10.1016/j.jim.2012.07.006 22841577

[116] Gomez-Roman VR Florese RH Patterson LJ Peng B Venzon D Aldrich K A simplified method for the rapid fluorometric assessment of antibody-dependent cell-mediated cytotoxicity. J Immunol Methods (2006) 308(1–2):53–67. 10.1016/j.jim.2005.09.018 16343526

[117] Pham TN Lukhele S Dallaire F Perron G Cohen EA. Enhancing virion tethering by BST2 sensitizes productively and latently HIV-infected T cells to ADCC mediated by broadly neutralizing antibodies. Sci Rep (2016) 6:37225. 10.1038/srep37225 27853288 PMC5112552

[118] Moldt B Schultz N Dunlop DC Alpert MD Harvey JD Evans DT A panel of IgG1 b12 variants with selectively diminished or enhanced affinity for Fcgamma receptors to define the role of effector functions in protection against HIV. J Virol (2011) 85(20):10572–81. 10.1128/JVI.05541-11 21849450 PMC3187489

[119] Ferrari G Pollara J Kozink D Harms T Drinker M Freel S An HIV-1 gp120 envelope human monoclonal antibody that recognizes a C1 conformational epitope mediates potent antibody-dependent cellular cytotoxicity (ADCC) activity and defines a common ADCC epitope in human HIV-1 serum. J Virol (2011) 85(14):7029–36. 10.1128/JVI.00171-11 21543485 PMC3126567

[120] Veillette M Coutu M Richard J Batraville LA Dagher O Bernard N The HIV-1 gp120 CD4-bound conformation is preferentially targeted by antibody-dependent cellular cytotoxicity-mediating antibodies in sera from HIV-1-infected individuals. J Virol (2015) 89(1):545–51. 10.1128/JVI.02868-14 25339767 PMC4301108

[121] Guan Y Pazgier M Sajadi MM Kamin-Lewis R Al-Darmarki S Flinko R Diverse specificity and effector function among human antibodies to HIV-1 envelope glycoprotein epitopes exposed by CD4 binding. Proc Natl Acad Sci U S A (2013) 110(1):E69–78. 10.1073/pnas.1217609110 23237851 PMC3538257

[122] Santra S Tomaras GD Warrier R Nicely NI Liao HX Pollara J Human non-neutralizing HIV-1 envelope monoclonal antibodies limit the number of founder viruses during SHIV mucosal infection in rhesus macaques. PLoS Pathog (2015) 11(8):e1005042. 10.1371/journal.ppat.1005042 26237403 PMC4523205

[123] Pollara J Bonsignori M Moody MA Pazgier M Haynes BF Ferrari G. Epitope specificity of human immunodeficiency virus-1 antibody dependent cellular cytotoxicity [ADCC] responses. Curr HIV Res (2013) 11(5):378–87. 10.2174/1570162X113116660059 24191939 PMC3878369

[124] Lewis GK Finzi A DeVico AL Pazgier M. Conformational masking and receptor-dependent unmasking of highly conserved Env epitopes recognized by non-neutralizing antibodies that mediate potent ADCC against HIV-1. Viruses (2015) 7(9):5115–32. 10.3390/v7092856 26393642 PMC4584300

[125] Wyatt R Moore J Accola M Desjardin E Robinson J Sodroski J. Involvement of the V1/V2 variable loop structure in the exposure of human immunodeficiency virus type 1 gp120 epitopes induced by receptor binding. J Virol (1995) 69(9):5723–33. 7543586 10.1128/jvi.69.9.5723-5733.1995 PMC189432

[126] Scheid JF Mouquet H Feldhahn N Seaman MS Velinzon K Pietzsch J Broad diversity of neutralizing antibodies isolated from memory B cells in HIV-infected individuals. Nature (2009) 458(7238):636–40. 10.1038/nature07930 19287373

[127] Mouquet H Scharf L Euler Z Liu Y Eden C Scheid JF Complex-type N-glycan recognition by potent broadly neutralizing HIV antibodies. Proc Natl Acad Sci U S A (2012) 109(47):E3268–77. 10.1073/pnas.1217207109 23115339 PMC3511153

[128] Mouquet H Klein F Scheid JF Warncke M Pietzsch J Oliveira TY Memory B cell antibodies to HIV-1 gp140 cloned from individuals infected with clade A and B viruses. PLoS One (2011) 6(9):e24078. 10.1371/journal.pone.0024078 21931643 PMC3169578

[129] Scheid JF Mouquet H Ueberheide B Diskin R Klein F Oliveira TY Sequence and structural convergence of broad and potent HIV antibodies that mimic CD4 binding. Science (2011) 333(6049):1633–7. 10.1126/science.1207227 21764753 PMC3351836

[130] Yasmeen A Ringe R Derking R Cupo A Julien JP Burton DR Differential binding of neutralizing and non-neutralizing antibodies to native-like soluble HIV-1 Env trimers, uncleaved Env proteins, and monomeric subunits. Retrovirology (2014) 11:41. 10.1186/1742-4690-11-41 24884783 PMC4067080

[131] Arias JF Heyer LN von Bredow B Weisgrau KL Moldt B Burton DR Tetherin antagonism by Vpu protects HIV-infected cells from antibody-dependent cell-mediated cytotoxicity. Proc Natl Acad Sci U S A (2014) 111(17):6425–30. 10.1073/pnas.1321507111 24733916 PMC4035966

[132] von Bredow B Arias JF Heyer LN Gardner MR Farzan M Rakasz EG Envelope glycoprotein internalization protects human and simian immunodeficiency virus-infected cells from antibody-dependent cell-mediated cytotoxicity. J Virol (2015) 89(20):10648–55. 10.1128/JVI.01911-15 26269175 PMC4580155

[133] Lu CL Murakowski DK Bournazos S Schoofs T Sarkar D Halper-Stromberg A Enhanced clearance of HIV-1-infected cells by broadly neutralizing antibodies against HIV-1 in vivo. Science (2016) 352(6288):1001–4. 10.1126/science.aaf1279 27199430 PMC5126967

[134] Guttman M Cupo A Julien JP Sanders RW Wilson IA Moore JP Antibody potency relates to the ability to recognize the closed, pre-fusion form of HIV Env. Nat Commun (2015) 6:6144. 10.1038/ncomms7144 25652336 PMC4338595

[135] Richard J Veillette M Ding S Zoubchenok D Alsahafi N Coutu M Small CD4 mimetics prevent HIV-1 uninfected bystander CD4+ T cell killing mediated by antibody-dependent cell-mediated cytotoxicity. EBioMedicine (2016) 3:122–34. 10.1016/j.ebiom.2015.12.004 26870823 PMC4739418

[136] Gohain N Tolbert WD Orlandi C Richard J Ding S Chen X Molecular basis for epitope recognition by non-neutralizing anti-gp41 antibody F240. Sci Rep (2016) 6:36685. 10.1038/srep36685 27827447 PMC5101508

[137] Richard J Veillette M Brassard N Iyer SS Roger M Martin L CD4 mimetics sensitize HIV-1-infected cells to ADCC. Proc Natl Acad Sci U S A (2015) 112(20):E2687–94. 10.1073/pnas.1506755112 25941367 PMC4443331

[138] Richard J Pacheco B Gohain N Veillette M Ding S Alsahafi N Co-receptor binding site antibodies enable CD4-mimetics to expose conserved anti-cluster A ADCC epitopes on HIV-1 envelope glycoproteins. EBioMedicine (2016) 12:208–18. 10.1016/j.ebiom.2016.09.004 27633463 PMC5078604

[139] Isitman G Lisovsky I Tremblay-McLean A Parsons MS Shoukry NH Wainberg MA Natural killer cell education does not affect the magnitude of granzyme B delivery to target cells by antibody-dependent cellular cytotoxicity. AIDS (2015) 29(12):1433–43. 10.1097/QAD.0000000000000729 26244383

[140] Chen Z Freedman MS. CD16+ gammadelta T cells mediate antibody dependent cellular cytotoxicity: potential mechanism in the pathogenesis of multiple sclerosis. Clin Immunol (2008) 128(2):219–27. 10.1016/j.clim.2008.03.513 18501678

[141] Braakman E van de Winkel JG van Krimpen BA Jansze M Bolhuis RL. CD16 on human gamma delta T lymphocytes: expression, function, and specificity for mouse IgG isotypes. Cell Immunol (1992) 143(1):97–107. 10.1016/0008-8749(92)90008-D 1377991

[142] Yeap WH Wong KL Shimasaki N Teo EC Quek JK Yong HX CD16 is indispensable for antibody-dependent cellular cytotoxicity by human monocytes. Sci Rep (2016) 6:34310. 10.1038/srep34310 27670158 PMC5037471

[143] Shaw GM Levy PC LoBuglio AF. Human monocyte antibody-dependent cell-mediated cytotoxicity to tumor cells. J Clin Invest (1978) 62(6):1172–80. 10.1172/JCI109236 748372 PMC371881

[144] Lanier LL Ruitenberg JJ Phillips JH. Functional and biochemical analysis of CD16 antigen on natural killer cells and granulocytes. J Immunol (1988) 141(10):3478–85. 2903193

[145] Mandelboim O Malik P Davis DM Jo CH Boyson JE Strominger JL. Human CD16 as a lysis receptor mediating direct natural killer cell cytotoxicity. Proc Natl Acad Sci U S A (1999) 96(10):5640–4. 10.1073/pnas.96.10.5640 10318937 PMC21913

[146] Bonsignori M Pollara J Moody MA Alpert MD Chen X Hwang KK Antibody-dependent cellular cytotoxicity-mediating antibodies from an HIV-1 vaccine efficacy trial target multiple epitopes and preferentially use the VH1 gene family. J Virol (2012) 86:11521–32. 10.1128/JVI.01023-12 22896626 PMC3486290

[147] Isitman G Tremblay-McLean A Lisovsky I Bruneau J Lebouche B Routy JP NK cells expressing the inhibitory killer immunoglobulin-like receptors (iKIR) KIR2DL1, KIR2DL3 and KIR3DL1 are less likely to be CD16+ than their iKIR negative counterparts. PLoS One (2016) 11(10):e0164517. 10.1371/journal.pone.0164517 27732638 PMC5061331

[148] O’Connor GM Vivian JP Widjaja JM Bridgeman JS Gostick E Lafont BA Mutational and structural analysis of KIR3DL1 reveals a lineage-defining allotypic dimorphism that impacts both HLA and peptide sensitivity. J Immunol (2014) 192(6):2875–84. 10.4049/jimmunol.1303142 24563253 PMC3948114

[149] Chung AW Navis M Isitman G Wren L Silvers J Amin J Activation of NK cells by ADCC antibodies and HIV disease progression. J Acquir Immune Defic Syndr (2011) 58(2):127–31. 10.1097/QAI.0b013e31822c62b9 21792067 PMC3175260

[150] Hidajat R Xiao P Zhou Q Venzon D Summers LE Kalyanaraman VS Correlation of vaccine-elicited systemic and mucosal nonneutralizing antibody activities with reduced acute viremia following intrarectal simian immunodeficiency virus SIVmac251 challenge of rhesus macaques. J Virol (2009) 83(2):791–801. 10.1128/JVI.01672-08 18971271 PMC2612365

[151] Burton DR Hessell AJ Keele BF Klasse PJ Ketas TA Moldt B Limited or no protection by weakly or nonneutralizing antibodies against vaginal SHIV challenge of macaques compared with a strongly neutralizing antibody. Proc Natl Acad Sci U S A (2011) 108(27):11181–6. 10.1073/pnas.1103012108 21690411 PMC3131343

[152] Dugast AS Chan Y Hoffner M Licht A Nkolola J Li H Lack of protection following passive transfer of polyclonal highly functional low-dose non-neutralizing antibodies. PLoS One (2014) 9(5):e97229. 10.1371/journal.pone.0097229 24820481 PMC4018276

